# Adult Neurogenesis in Neurodegenerative Diseases: Mechanisms of Dysregulation in Alzheimer’s and Parkinson’s Disease

**DOI:** 10.3390/ijms27062742

**Published:** 2026-03-17

**Authors:** Magdalena Dębiec, Marcin Rojek

**Affiliations:** 1 Department of Physiology, Faculty of Medical Sciences in Katowice, Medical University of Silesia, 40-055 Katowice, Poland; 2 Department of Biophysics, Faculty of Medical Sciences in Zabrze, Medical University of Silesia, 40-055 Katowice, Poland; 3 Department of Histology and Cell Pathology, Faculty of Medical Sciences in Zabrze, Medical University of Silesia, 40-055 Katowice, Poland

**Keywords:** neurogenesis, neurodegenerative diseases, Alzheimer’s disease, Parkinson’s disease, inflammation, aging, inflammasome

## Abstract

Adult neurogenesis, the process of generating new, functional neurons in the mature central nervous system, represents a key mechanism of brain plasticity and a potential source of regeneration. This process occurs primarily within specialised neurogenic niches: the subgranular zone of the hippocampal dentate gyrus (SGZ) and the subependymal zone (SEZ). It is regulated by a complex network of endogenous factors (e.g., hormones, neurotrophins, growth factors) and exogenous factors (environment, stress, diet, physical activity). Impairments in neurogenesis are linked to the pathogenesis of neurodegenerative diseases, such as Alzheimer’s disease (AD) and Parkinson’s disease (PD). In their course, chronic inflammation, mitochondrial dysfunction, oxidative stress, and the accumulation of pathological proteins (β-amyloid, Tau protein, α-synuclein) create a microenvironment that inhibits the proliferation, differentiation, and survival of new neurons. This results in the exacerbation of cognitive and memory deficits. A review of the literature indicates that modulating neurogenesis through non-pharmacological interventions (e.g., a diet rich in anti-inflammatory compounds, physical exercise) and targeted therapeutic strategies represents a promising, albeit complex, research avenue. The primary challenge remains not only stimulating neuron generation but also ensuring their proper maturation, survival, and functional integration into existing neuronal circuits. A deeper understanding of the molecular and environmental mechanisms regulating adult neurogenesis may open new therapeutic possibilities for slowing the progression of neurodegenerative diseases.

## 1. Introduction

Neurogenesis is the process that leads to the formation of new, functional neurons from precursor cells, neuroblast-derived from the neural system’s stem cells and glial cells. It encompasses both prenatal (embryonic) and postnatal neurogenesis, the latter occurring also in the brains of adult individuals [1].

This process constitutes one of the fundamental biological determinants of the regenerative capacity of the central nervous system. A pivotal moment in the research on adult brain neurogenesis was the isolation in 1995 by Palmer of hippocampal progenitor cells based on their morphological features and the expression of characteristic markers [2]. The discovery of neural stem cells (NSCs) not only confirmed the existence of neurogenesis in adult mammals, but also initiated intensive research into the mechanisms regulating this process and its potential application in regenerative strategies for the nervous system [1].

In adult individuals, neurogenesis occurs primarily in specialized neurogenic niches, including the dentate gyrus of the hippocampus—a structure playing a key role in spatial and episodic memory processes. Due to the hippocampus’s fundamental significance for learning, memory consolidation, and cognitive adaptation, this region remains one of the best-understood brain areas in the context of adult neurogenesis [2].

Neurogenesis is a multi-stage process encompassing the proliferation, differentiation, and maturation of nerve cells. Neural stem cells play a key role in this process, characterized by their capacity for self-renewal and the generation of progeny cells with progressively limited proliferative potential. In the first stage, neural stem/progenitor cells (NSPCs) are formed, which exhibit a directed tendency to differentiate into specific cell lineages. In the subsequent course of the process, NSPCs transform into neuroblasts, and then into mature neurons or glial cells, such as astrocytes and oligodendrocytes [3].

The process of neurogenesis occurs within a strictly defined microenvironment, referred to as a stem cell niche, which regulates the proliferation, differentiation, cell fate, and survival of progeny cells. In the developing brain, the primary sites of active neurogenesis are the ventricular zone (VZ) and the subventricular zone (SVZ). Intensive divisions of neural stem cells (NSCs) and progenitor cells in these regions lead to the formation of the majority of neurons and a significant portion of glial cells in the central nervous system. As the brain matures, its neurogenic potential becomes significantly restricted. In adult individuals, it persists only within specialized neurogenic niches, such as the subependymal zone (SEZ, corresponding to the adult SVZ) and the subgranular zone (SGZ) of the hippocampal dentate gyrus. In these areas, neurogenesis occurs at a much lower level and primarily serves adaptive and plastic functions [4].

Progenitor cell niches constitute specialized microenvironments that play a key role in initiating and modulating active neurogenesis. The concept of a stem cell niche was proposed in 1978 by Schofield, while its functional description was first presented by Xie and Spradling in 2000. Although stem cell niches in tissues such as bone marrow, intestine, liver, or skin have been well characterized, the structure and function of neural stem cell (NSC) niches remain incompletely understood. Currently, six main cell types that build NSC niches are distinguished: vascular endothelial cells, ependymal cells, astrocytes, microglial cells, neural stem cells, and mature neurons. Components of the extracellular matrix also play a significant role, providing the structural scaffold of the niche and participating in the spatial organization of its elements. Through paracrine signaling and direct cell–cell interactions, NSC niches create a dynamic functional environment, capable of adapting in response to changes in the organism’s homeostatic conditions [5].

The production of new neurons is a lifelong process in many species and constitutes an essential mechanism for maintaining brain plasticity. Neurogenesis in the adult central nervous system is often viewed as a process sustaining the pool of stem cells originating from the embryonic period. These cells exhibit similarity to radial glia and are termed radial glia-like cells (RGLs). Analysis of their phenotype, however, indicates that they are not merely residual developmental precursors. Compared to embryonic radial glial cells, adult RGLs are characterized by an extended cell cycle, more restricted multipotency, and distinct transcriptomic programs [5].

**Figure 1:**
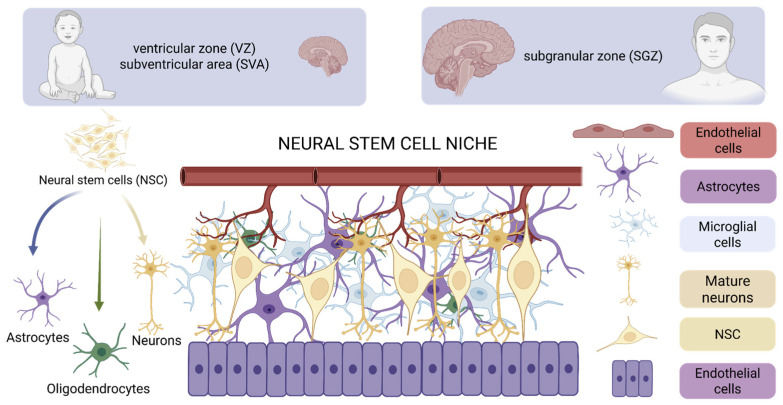
Neural stem cell niches in the developing and mature brain. Created in Biorender. Dębiec, M.; Rojek, M. (2026) https://biorender.com/x48hi1o.

In the mature brain, neural stem cells are primarily located in the subgranular zone of the hippocampal dentate gyrus and in the subependymal zone of the lateral ventricles (Figure 1). NSCs present in the SGZ generate new neurons that are incorporated locally into hippocampal circuits, while cells originating from the SEZ migrate along the rostral migratory stream to the olfactory bulb, where they differentiate mainly into interneurons (Figure 1). Besides the cellular components of the niche, cerebrospinal fluid (CSF) also plays a significant regulatory role within the SEZ. The niche located in the SGZ is primarily constructed by astrocytes and vascular endothelium, which together participate in controlling NSC proliferation and differentiation [6].

The cellular components of the neurogenic niche interact with neural stem cells by activating specific signaling pathways, leading to changes in the cells’ biochemical profile. The effect of these interactions is the regulation of specific gene expression, and consequently, the determination of cell fate and the shaping of the phenotype of progeny cells.

Disturbances in neurogenesis processes are associated with a broad spectrum of central nervous system pathologies, including cognitive dysfunctions and the development of neurodegenerative diseases. Among the most frequently mentioned disease entities are Alzheimer’s disease, Parkinson’s disease, and Huntington’s disease, as well as psychiatric disorders such as depression and anxiety disorders [7].

## 2. Methods of Literature Review

A literature review was conducted based on an analysis of the most recent scientific reports on adult neurogenesis, with particular emphasis on Parkinson’s disease and Alzheimer’s disease. Relevant publications were identified through searches of the MEDLINE and Google Scholar databases. The search strategy was based on combinations of keywords related to adult neurogenesis and neurodegenerative diseases (e.g., adult neurogenesis, Parkinson’s disease, Alzheimer’s disease, neural stem cells, hippocampal neurogenesis).

The review included original research articles and review papers. Conference presentations were excluded from the analysis if the full text of the contribution was not available. Eligibility criteria required access to the full-text publication.

Given the nature of the field, foundational and conceptual studies were also included in the final analysis. Overall, the publication timeframe spanned from 1993 to 2025. Older studies, despite their limited relevance to the most recent findings, were incorporated to preserve the historical context of the development of research on adult neurogenesis.

## 3. Neurogenesis Regulation

### 3.1. Factors Influencing Neurogenesis

Neurogenesis is regulated by numerous endogenous and exogenous factors, which directly or indirectly influence the individual stages of this process, including the proliferation of neural stem cells, the differentiation of progeny cells, and their survival. Hormones, particularly sex hormones and thyroid hormones, play a significant modulating role.

Experimental studies indicate a significant influence of estrogens on neurogenesis in the dentate gyrus of the hippocampus, which is important for learning and memory processes. However, this effect is transient and depends on hormone concentration, and thus on the phase of the sexual cycle. Estrogens affect not only the proliferation of progenitor cells but also the survival of newly formed neurons. It has been demonstrated that estradiol administration leads to an increase in the total number of newly generated cells, indicating an improvement in their survival rate. At the same time, impairments in cognitive functions, including deterioration of learning and spatial memory, were observed [8,9].

The influence of estrogen deficiency on neurogenesis was evaluated in animal models using bilateral ovariectomy. In adult female rats subjected to this procedure, a rapid decrease in serum estradiol concentration was observed within 24 h, accompanied by a reduction in cell proliferation and the number of newly formed immature neurons compared to the sham-operated control group [10,11]. Other studies demonstrated that local administration of estradiol increases hippocampal cell proliferation in 5-day-old female rats. However, observations indicating a low level or absence of estradiol in the hippocampus of adult rats 2–3 weeks after ovariectomy challenge the hypothesis of a permanent, local action of this hormone in the mature brain [12].

Testosterone also exhibits a stimulatory effect on neurogenesis [13]. Studies conducted on female and male rats demonstrated a higher survival rate of newly formed cells in the dentate gyrus in males compared to females [14,15,16]. At the same time, no significant sex-dependent differences in cell proliferation in this hippocampal region were observed [13].

Thyroid hormones play a complex regulatory role in the central nervous system, exerting inhibitory effects on neurogenesis, neuronal migration, dendritic development, and synaptogenesis, as well as modulating neurotransmitter release and function. The most extensive data on their action come from animal models of thyroid diseases [17]. It has been shown that thyroid hormones regulate neuronal migration within the cerebral cortex, hippocampus, and cerebellum, while their deficiency during development leads to disturbances in the organization of cortical layers [18,19,20].

Factors that lower the rate of neurogenesis also include chronic sleep deprivation and elevated glucocorticoid levels, most often associated with prolonged stress [21]. Stress exerts a negative impact on neurogenic niches, particularly in brain areas involved in emotion and memory regulation. Both exposure to stress and the chronic anticipation of stressful situations lead to the inhibition of neurogenesis in the hippocampus. Chronic stress additionally induces changes in the dendritic morphology of hippocampal neurons, which is associated with a reduction in the volume of this structure [22].

The neurogenesis process also undergoes significant weakening with age. In the aging brain, a decrease in progenitor cell proliferation, a reduction in the survival of newly formed cells, and a limitation of their migration are observed, resulting in a lowered number of neurons achieving full functional maturity [23].

Disturbances in neurogenesis are associated with the development of depression and a decline in cognitive functions. A growing body of evidence indicates that lifestyle modifications can promote the maintenance of the neural stem cell pool under conditions conducive to their proliferation and survival, with particular importance attributed to dietary factors. It has been demonstrated that many adverse changes affecting neurogenesis are interconnected and largely stem from chronic, low-grade inflammation persisting in the central nervous system. Reducing inflammatory processes may therefore contribute to the partial restoration of nerve cell renewal capacity.

Experimental studies have provided evidence for the beneficial effects of specific nutrients on stimulating neurogenesis. Among the best-characterized are curcumin [24], omega-3 polyunsaturated fatty acids [25], polyphenols (including those present in berries) [26], and flavanols, a significant source of which is cocoa (*Theobroma cacao*) [27]. These compounds exhibit anti-inflammatory and antioxidant effects, which may indirectly support the maintenance of functional neurogenic niches.

In recent years, attention has also been drawn to the role of diet in modulating the composition of the gut microbiota and its indirect influence on brain function within the gut–brain axis. Deepening the understanding of interactions between the gut microbiota and neurogenic processes may open new therapeutic perspectives and constitute a promising complement to standard pharmacological strategies [28].

### 3.2. Factors Regulating and Modulating Neurogenesis

The molecular regulation of neural stem cell (NSC) development in the neurogenic niches of the subgranular zone (SGZ) of the dentate gyrus and the subventricular zone (SVZ) involves numerous signaling factors, including neurotransmitters, neuregulins, and growth factors. Key regulators include vascular endothelial growth factor (VEGF), epidermal growth factor (EGF), and fibroblast growth factor (FGF) [15].

Mature NSCs, due to their morphological phenotype, are designated as radial glia-like cells (RGLs). They are characterized by their capacity for self-renewal and multipotency, which enables the maintenance of a stable pool of stem cells within the neurogenic niche. The ability to undergo multiple divisions ensures the continuity of neurogenesis, while multipotency allows for the generation of phenotypically diverse progeny cells. Through successive divisions, RGLs can differentiate into glial cells or, after transitioning to a type II progenitor cell phenotype, enter the neuronal differentiation pathway [27].

**Figure 2:**
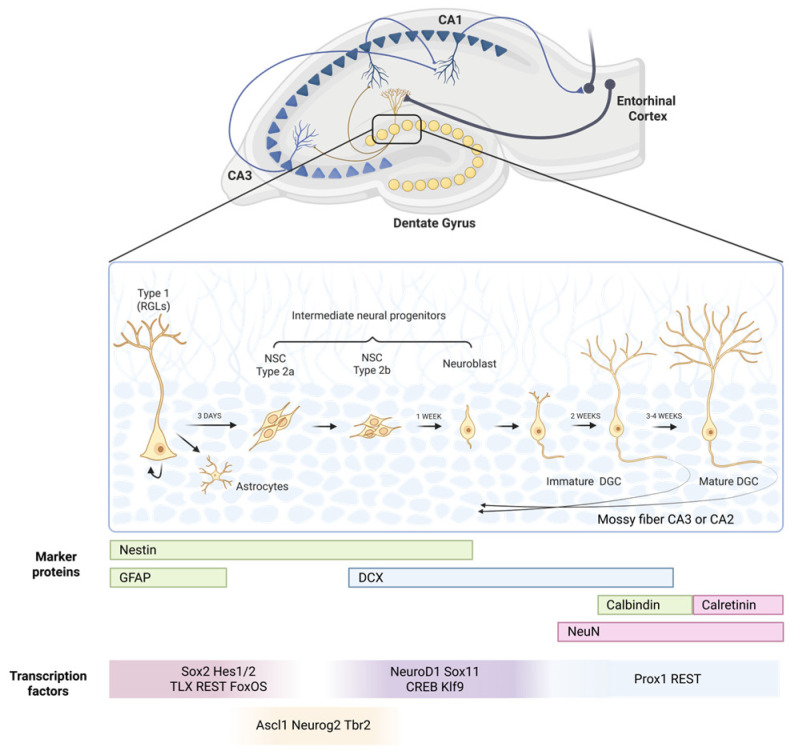
Stages of neurogenesis. Created in Biorender. Dębiec, M.; Rojek, M. (2026) https://biorender.com/oosao1h.

Type II progenitor cells are divided into subtypes IIa and IIb, which differ in their expression profiles of regulatory proteins, including the transcription factor *Sox2*. During further differentiation, these cells acquire the characteristics of neuroblasts, in which the expression of specific transcription factors, such as *NeuroD1*, *Tbr2*, or *Sox1*, directs subsequent development towards mature neurons [16] (Figure 2). Individual stages of neurogenesis can be identified based on the expression of specific protein markers, enabling their precise experimental distinction (Figure 2). In Figure 2, arrows indicate the sequential progression of adult hippocampal neurogenesis, from radial glia-like stem cells through intermediate progenitors and neuroblasts to immature and mature dentate granule cells.

Transcription factors play a key role in regulating gene expression and constitute important mediators of signals originating from the neurogenic niche environment. Their sequential and coordinated expression is essential for the proper progression from neural stem cells to functionally mature neurons (Figure 1). Type II progenitors, characterized by an irregular shape, express nestin and/or doublecortin (DCX) (Figure 2). Conversely, NeuN (neuronal nuclear protein) is a marker specific for mature neurons, present exclusively in nervous tissue and not expressed by glial cells [17].

In comparison to the brain during the embryonic development period, the mature brain is characterized by a highly organized architecture of neural circuits. The hippocampal dentate gyrus (DG) receives intensive glutamatergic projections from the entorhinal cortex via the perforant path, as well as local input signals originating from several populations of GABAergic interneurons. Furthermore, the DG is modulated by numerous neuromodulatory systems, including cholinergic and GABAergic projections from the septal nuclei, glutamatergic inputs from the supramammillary area, serotonergic projections from the raphe nuclei, noradrenergic inputs from the locus coeruleus, and dopaminergic projections from the ventral tegmental area.

An important factor influencing the course of neurogenesis is the environment in which the organism functions. It has been demonstrated that in animals maintained in enriched environments, both under natural and experimental conditions characterized by increased stimulation of visual, olfactory, and taste stimuli, a significant increase in the generation and survival of newly formed neurons in the hippocampus is observed [15].

Some endogenous factors also exert a positive influence on the neurogenesis process, particularly neurotrophins and growth factors, such as nerve growth factor (NGF), brain-derived neurotrophic factor (BDNF), neurotrophin 3 (NT-3), and neurotrophin 4/5 (NT-4/5). These compounds exhibit distinct biological properties and selectively interact with specific populations of nerve cells. Additionally, some neurotransmitters can modulate neurogenesis by stimulating cell proliferation within neurogenic niches [20].

BDNF is among the best-characterized neurotrophic factors and plays a key role in regulating the differentiation, maturation, and survival of neurons in the central nervous system. It also exhibits neuroprotective action under adverse conditions, such as excessive glutamatergic stimulation, cerebral ischemia, hypoglycemia, or exposure to neurotoxic factors [21]. BDNF actively participates in the control of neurogenesis, and its protein and mRNA have been detected in most brain structures, including the olfactory bulb, cerebral cortex, hippocampus, basal forebrain, midbrain, hypothalamus, brainstem, and spinal cord. Beyond neurotrophic functions, BDNF also plays a significant role in regulating energy homeostasis. It has been demonstrated that BDNF administration, both peripherally and intracerebroventricularly (ICV), leads to reduced energy intake and body weight. At the same time, decreased BDNF levels are observed in the course of neurodegenerative diseases, such as Parkinson’s disease, Huntington’s disease, and multiple sclerosis [22].

## 4. Inflammation-Driven Disruption of Neurogenesis in Aging and Parkinsonian Neurodegeneration

The intensification of inflammatory processes in the adult brain constitutes one of the key factors disrupting neurogenesis. In the course of chronic inflammation, metabolic and circulatory disturbances occur, which favor the accumulation of toxic metabolic products in the brain parenchyma, leading to impaired function of neural structures [29]. A central element of neuroinflammation is the activation and accumulation of microglial cells, which, under conditions of chronic pro-inflammatory stimulation, exhibit neurotoxic effects. Factors contributing to the persistence of chronic inflammation in the brain also include comorbidities such as arterial hypertension, diabetes, and obesity [30].

In elderly individuals, especially those burdened with chronic diseases, elevated levels of peripheral pro-inflammatory cytokines are observed, including interleukin 6 (IL-6), tumor necrosis factor alpha (TNF-α), and C-reactive protein. Elevated IL-6 levels have been associated with cognitive function decline, reduced physical fitness, and an increased risk of developing age-related pathologies [31]. However, it should be emphasized that an increase in IL-6 concentration is also observed in the plasma of aging individuals without clinical symptoms of disease, indicating the complex and multifactorial nature of inflammatory processes accompanying organismal aging [32].

Increasing attention is being paid to the role of the gut microbiota as a factor modulating chronic inflammation in the course of brain aging. Changes in the composition of the gut flora can lead to increased production of pro-inflammatory cytokines, contributing to the accelerated development of vascular pathologies, including ischemic stroke [33]. At the same time, stroke itself induces significant changes in the composition of the gut microbiota, and interactions within the gut–brain axis play a significant role in the progression of this condition [34]. A growing number of studies indicate that the gut microbiota is also involved in the pathogenesis of numerous neurodegenerative and neuropsychiatric diseases, highlighting the significance of the gut–brain axis in aging processes and its potential role in the development of age-related neurodegenerative diseases [35].

Inflammatory and infectious processes accompanying neurodegenerative diseases, such as Parkinson’s disease, exert a significant negative impact on hippocampal neurogenesis. In animal models, this includes, among other things, a reduction in the number of newly formed neurons, assessed based on BrdU/NeuN double-positive cells in the dentate gyrus [36,37]. Symptoms observed in patients with Parkinson’s disease and other neurodegenerative diseases, including cognitive and memory function impairments, may be at least partially a consequence of reduced hippocampal neurogenesis [38]. Therefore, a hypothesis has been proposed that restoring or modulating hippocampal neurogenesis may have clinical significance in alleviating cognitive deficits [39]. It should be noted, however, that the direct mechanisms responsible for the decline in cognitive function in the course of neurodegenerative diseases, as well as the precise role of hippocampal neurogenesis in shaping neurological and mental well-being, remain incompletely understood [38].

Neuroinflammation plays a significant role in the worsening of cognitive functions in both Alzheimer’s disease and Parkinson’s disease, making it a potential target for new therapeutic strategies. Excessive activation of microglia promotes neurodegeneration, while inflammatory markers are increasingly being considered as potential prognostic biomarkers. It has been demonstrated that the concentrations of pro-inflammatory cytokines in the cerebrospinal fluid correlate with the severity of cognitive impairments in patients with Parkinson’s disease [40].

## 5. Neurogenesis, Aging, and Neuroinflammation

The mobilization of endogenous neural stem cells (NSCs) is intensively investigated as a potential regenerative strategy aimed at restoring lost brain functions in the course of cerebrovascular diseases, traumatic brain injuries, and neurodegenerative diseases. Despite promising preclinical results, this approach is associated with numerous limitations, including the risk of depleting the NSC pool, the limited survival capacity of newly formed neurons, and the lack of precise control over their targeted migration and integration with existing neuronal circuits.

Identifying the molecular and environmental mechanisms regulating adult neurogenesis, as well as the factors responsible for its physiological decline associated with aging, may in the future enable the development of more targeted and safer therapeutic strategies [41].

### 5.1. Neurogenesis in Alzheimer’s Disease

Alzheimer’s disease (AD) is the most prevalent neurodegenerative disease worldwide and the leading cause of dementia. According to data from the National Health Fund from 2022, the number of people suffering from Alzheimer’s disease worldwide is currently about 39 million. Due to the aging population, it is projected that by 2050 this number will increase to about 152.8 million, making AD one of the most serious public health challenges of the 21st century [42]. The clinical picture of the disease includes progressive deterioration of memory and executive functions, language and visuospatial disturbances, as well as changes in patients’ behavior and personality [43].

A growing body of evidence indicates that neurogenesis in the brains of adults suffering from AD undergoes significant disturbances. Studies conducted on animal models suggest that modulating neurogenic processes may influence the course of the disease, making neurogenesis a potential therapeutic target. Degeneration of neuronal projections terminating in the hippocampal dentate gyrus is associated with increased neuronal death in AD, which further disrupts the functioning of hippocampal circuits [44]. It has also been demonstrated that regular physical activity of varying intensity can stimulate hippocampal neurogenesis and potentially reduce the risk of developing Alzheimer’s disease [45]. One of the early pathogenic mechanisms of AD is mitochondrial dysfunction, which co-occurs with the accumulation of insoluble amyloid-β (Aβ) deposits and intracellular neurofibrillary tangles composed of hyperphosphorylated Tau protein. These changes lead to disturbances in synaptic transmission and gradual neuronal loss [46]. Numerous studies confirm the key role of Tau protein in the development and progression of Alzheimer’s disease [47].

The process of mitophagy, regulated among others by the *PINK1*/Parkin pathway, can modulate Tau pathology. In experimental studies, including those using a *Caenorhabditis elegans* nematode model with a Tau mutation, it has been demonstrated that disruption of proper Parkin translocation to mitochondria leads to mitophagy dysfunction and intensification of neuronal pathology [48,49]. At the same time, a growing number of reports indicate that modulating the function of the Tau protein may constitute a promising therapeutic direction in AD and related diseases [50,51,52]. In the pathogenesis of neurogenesis disturbances in AD, neurotransmitter systems, particularly the glutamatergic and cholinergic systems, also play a significant role. Studies on animal models have shown that the inhibition of glutamatergic signaling leads to a reduction in cell proliferation in the hippocampal neurogenic niches [53]. On the other hand, acetylcholine promotes the formation of new neurons in the dentate gyrus, which is reflected in therapeutic strategies based on acetylcholinesterase inhibitors used in the symptomatic treatment of AD [54]. Disturbances in cholinergic transmission may also favor the abnormal phosphorylation of the Tau protein and the intensification of inflammatory processes within the nervous system, although the precise mechanisms of these relationships remain unclear. Due to the multifactorial and complex pathogenesis of Alzheimer’s disease, effective methods for prevention and causal treatment remain currently unavailable, and intensive research into new therapeutic strategies is being conducted worldwide [55].

### 5.2. Neurogenesis in Parkinson’s Disease

Parkinson’s disease (PD) is a progressive, chronic neurodegenerative disease, first described in 1817 by James Parkinson. This condition most commonly affects elderly individuals, and its incidence significantly increases after the age of 65, reaching a rate of 4–5% of the population in individuals over 85 years old [56]. The pathophysiological basis of Parkinson’s disease is progressive damage to the extrapyramidal system, which clinically manifests as bradykinesia, muscle rigidity, resting tremor, and disturbances in gait and posture. However, PD is now understood as a multifaceted disorder with a wide range of clinical symptoms that extend beyond the classical motor dysfunctions. Notably, patients often suffer from non-motor symptoms, including cognitive deficits such as dementia, as well as autonomic and gastrointestinal disturbances [56].

The etiology of Parkinson’s disease is complex and encompasses both genetic and environmental factors. In some patients, hereditary forms of the disease have been identified, associated with mutations in genes encoding, among others, *PINK1* kinase, parkin, ubiquitin hydrolase L1, and α-synuclein [57]. In autosomal recessive early-onset forms of PD, mutations in the *PINK1*, *PARK2*, and *PARK6* genes are particularly common. In patients with the *PARK2* mutation, as well as in studies conducted on murine models with this mutation, increased expression of the NLRP3 inflammasome in microglial cells and macrophages has been observed. This mechanism is associated with impaired expression of the anti-inflammatory protein A20, which under physiological conditions inhibits NLRP3 inflammasome activation [58]. However, it should be emphasized that most data regarding the role of the NLRP3 inflammasome come from in vitro studies, and its pathogenic significance in Parkinson’s disease remains incompletely understood.

It has been demonstrated that α-synuclein can activate the NLRP3 inflammasome in human monocytes and in BV2 microglial cell lines; however, this effect has not been unequivocally confirmed in primary microglial cultures [59]. Besides the pathological accumulation of α-synuclein, key factors leading to the loss of dopaminergic neurons in Parkinson’s disease include mitochondrial dysfunction and oxidative stress, although the precise mechanisms of these processes have not yet been fully elucidated [60].

Increasing interest is being focused on the potential role of adult neurogenesis as a compensatory mechanism that could limit the loss of dopaminergic neurons in the midbrain. Experimental studies suggest that stimulation of neurogenesis may reduce the severity of dyskinesias and slow the progression of neurodegeneration in Parkinson’s disease [61,62].

Neurodegeneration induced by inflammatory processes in PD is characterized by delayed, yet progressive, neuronal loss. Neuronal damage and paracrine and autocrine signals, including cytokines and reactive oxygen species (ROS), lead to the activation of microglia, which can remain in a state of chronic activation even after the primary stimulus subsides. Long-term activated microglia become a source of pro-inflammatory cytokines and free radicals, intensifying neurotoxicity and contributing to the progression of Parkinson’s disease. The microglial response to neuronal damage and oxidative stress is currently considered one of the key mechanisms of chronic neuroinflammation in PD [63].

Epidemiological studies have observed that the use of non-steroidal anti-inflammatory drugs is associated with a reduced risk of developing Parkinson’s disease, which may indicate a potential neuroprotective effect of modulating inflammatory processes [64]. At the same time, the importance of optimizing symptomatic treatment is emphasized. A priority for future clinical research remains the evaluation of whether modern strategies ensuring stable plasma levodopa concentrations, implemented in the early stages of the disease, can delay the onset of drug-induced dyskinesias [65]. Similar challenges in understanding disease etiology apply to Alzheimer’s disease (AD), where the core pathophysiological mechanisms remain elusive. Although beta-amyloid and phosphorylated Tau are central to AD research, they are insufficient to fully explain disease onset and progression. Consequently, increasing attention has been directed toward novel biomarkers, including phosphoglycerate dehydrogenase, clusterin, microRNAs, novel peptide ratios (Aβ37/Aβ42), plasma glial fibrillary acidic protein, and lipid peroxidation markers, as well as toward neurovascular unit dysfunction and emerging diagnostic and therapeutic innovations [66]. Beyond classical neurodegenerative pathways, the microbiome has emerged as a potential modifier of brain aging. Growing evidence indicates its involvement in cognitive decline; however, the underlying mechanisms remain incompletely understood. Age-related alterations in the gut microbiome and immune system are thought to be closely interconnected, with immune dysfunction representing a key pathway linking microbiome changes to brain function. Disruption of immunological homeostasis may promote neuroinflammation and blood–brain barrier dysfunction, thereby contributing to cognitive decline. Nevertheless, comprehensive knowledge regarding specific microbiome–immune interactions that influence neuronal and cognitive health during aging is still limited. Recent reviews have summarized current evidence on microbiome profiles associated with healthy versus unhealthy aging and examined how these alterations interact with immune cells involved in neuronal and cognitive processes, as well as the potential role of microbiome-modulating interventions such as probiotics, prebiotics, and postbiotics in preserving cognitive function in older adults [67]. Beyond classical biomarkers and immune–microbiome interactions, emerging evidence points to the involvement of developmental signalling pathways in neurodegeneration. Recent findings highlight the role of Sonic hedgehog (Shh) signalling in Alzheimer’s disease pathogenesis, with its disruption linked to impaired neurogenesis, blood–brain barrier dysfunction, and increased vulnerability to amyloid beta toxicity. Notably, several traditional remedies used in AD may exert neuroprotective effects by stimulating this pathway [68].

## 6. Conclusions

Neurogenesis, primarily associated with the prenatal period and early youth, is increasingly gaining interest in the context of its role in the plasticity of the mature central nervous system. As demonstrated in this paper based on a review of the current literature, the subgranular zone of the hippocampal dentate gyrus and the subependymal zone of the lateral ventricles constitute specialized cellular niches where a precise balance between trophic factors, paracrine signals, and microenvironment components determines the proliferation, differentiation, and survival of new nerve cells.

The process of adult neurogenesis is subject to complex and multi-level regulation. Among the identified factors influencing its intensity, endogenous factors should be mentioned, including hormones (estrogens, testosterone, thyroid hormones) and neurotrophins (particularly BDNF), as well as exogenous factors such as stress, diet, physical activity, and living environment. These elements contribute to a homeostatic balance whose disruption can affect the brain’s regenerative and compensatory capacities.

**Figure 3:**
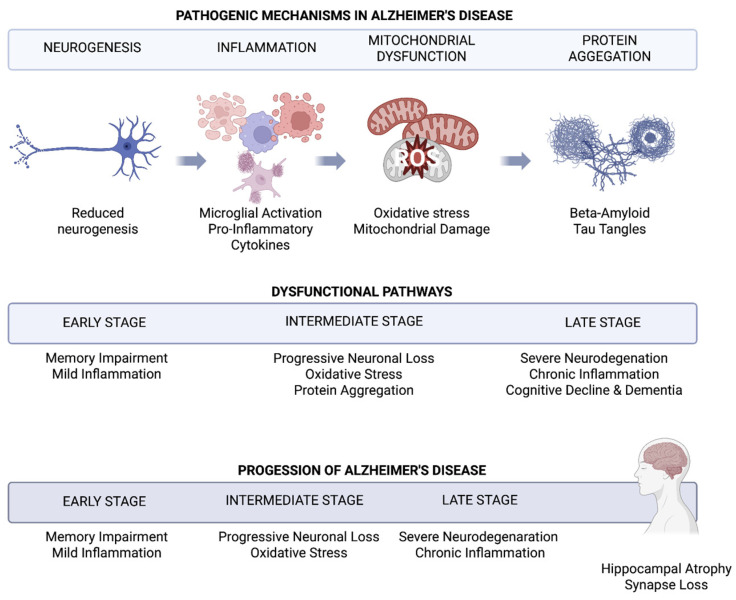
The Interplay of Inflammation, Protein Aggregation, and Neurogenesis in the Progression of Alzheimer’s Disease. Created in Biorender. Dębiec, M.; Rojek, M. (2026) https://biorender.com/chrv840.

The analysis of the relationship between adult neurogenesis and Alzheimer’s disease and Parkinson’s disease points to a shared pathogenic basis. Both diseases are characterized by a common pool of neurodegenerative mechanisms, including chronic microglial activation, oxidative stress, and disturbances in signaling pathways. These factors translate into the maintenance of a brain microenvironment that inhibits neurogenesis and the brain’s regenerative capabilities. The resulting impairment of hippocampal neurogenesis has been linked to broader cognitive dysfunction observed in patients. Figure 3 illustrates this relationship by showing how the three core pathological mechanisms, namely inflammation, protein aggregation (beta-amyloid and tau tangles), and reduced neurogenesis, interact and drive the progression of Alzheimer’s disease from early memory impairment to severe neurodegeneration and cognitive decline.

**Figure 4:**
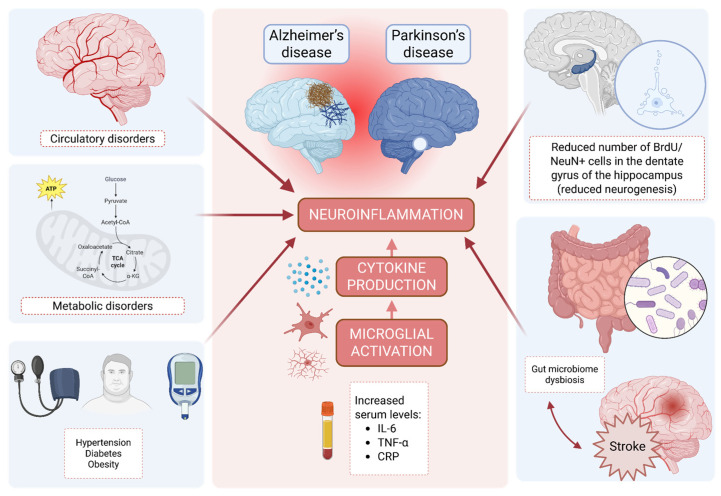
Pathophysiological Pathways Connecting Comorbidities, Gut Dysbiosis, and Neuroinflammation. Created in Biorender. Dębiec, M.; Rojek, M. (2026) https://biorender.com/si36u8w.

Despite the identification of potentially beneficial therapeutic avenues, a closer examination of the literature reveals the daunting complexity inherent in translating adult neurogenesis into clinical practice. The principal impediments stem from the characteristically low survival rate of nascent neurons in the pathological microenvironment and the subsequent challenges in achieving their precise functional integration. For any therapeutic benefit to be realized, these new cells must not only survive but also establish appropriate synaptic connections, effectively replacing neurons within pre-existing, often disrupted, neural circuits. The failure to surmount these biological hurdles largely accounts for the lack of translatable success observed in clinical trials to date. Consequently, the primary objective in mobilizing endogenous stem cell pools shifts from merely stimulating progenitor proliferation to the more formidable task of ensuring the long-term viability and seamless functional engraftment of their progeny into established hippocampal or subventricular networks. The complex interactions between systemic comorbidities, gut microbiota alterations, microglial activation, and cytokine production contributing to neuroinflammation and impaired neurogenesis are schematically illustrated in Figure 4.

Inflammation-Driven Disruption of Neurogenesis in Aging and Parkinsonian Neurodegeneration.

In summary, adult neurogenesis, with successive scientific reports, is ceasing to be merely a concept and has become an established pillar of brain plasticity. Its deeper understanding, particularly in the context of interactions with inflammatory and metabolic processes, opens new potential pathways for slowing the progression of neurodegenerative diseases and improving the quality of life for the aging population. At the same time, attention should be drawn to the limited scale of impact among the factors already identified as allowing influence on neurogenesis in adult animals and humans. Further research should focus on developing safe and effective methods for the targeted modulation of this process in strictly defined pathological contexts.

It is also worth noting that future efforts to summarize advancements in this field should encompass a broader range of scientific evidence, including additional databases, as well as patents and preprints.

## Data Availability

No new data were created or analyzed in this study. Data sharing is not applicable to this article.
